# Genome Sequence of Highly Pathogenic Avian Influenza Virus A/Chicken/North Kazakhstan/184/2020 (H5N8)

**DOI:** 10.1128/mra.01151-22

**Published:** 2023-05-08

**Authors:** B. Baikara, A. Seidallina, B. Baimakhanova, Y. Kasymbekov, T. Sabyrzhan, K. Daulbaeva, S. Nuralibekov, Y. Khan, K. Karamendin, A. Sultanov, A. Kydyrmanov

**Affiliations:** a Al-Farabi Kazakh National University, Almaty, Kazakhstan; b Kazakh Scientific Research Veterinary Institute, Almaty, Kazakhstan; c Research and Production Center for Microbiology and Virology, Almaty, Kazakhstan; Portland State University

## Abstract

The influenza virus strain A/chicken/North Kazakhstan/184/2020 (H5N8) was isolated in North Kazakhstan during a highly pathogenic avian influenza outbreak in 2020. This study aimed to obtain the complete genome sequence of the isolate.

## ANNOUNCEMENT

Influenza A viruses are enveloped single-stranded RNA viruses, with a segmented negative-sense genome, that belong to the family *Orthomyxoviridae* and genus *Alphainfluenzavirus* (1). Highly pathogenic avian influenza (HPAI) viruses continue to circulate in avifauna and periodically spill over to domestic birds and commercial flocks (2). HPAI with hemagglutinin (HA) of subtype H5 has significant epidemic potential (3).

Tracheal and cloacal swabs were taken from affected chickens in the North Kazakhstan Region (53°52′48.0″N, 67°24′00.0″E) during an outbreak of avian influenza (4). The sample was positive by PCR for the HA and M genes, and subsequently, H5N8 influenza A virus was isolated from embryonated chicken eggs (5).

For genomic analysis, viral RNA was extracted from the allantoic fluid using the QIAamp viral RNA minikit (Qiagen, Germany) according to the manufacturer’s manual. To amplify the whole influenza A genome, 14 pairs of primers were used (6). All eight gene segments were amplified, with 2 to 3 overlapping PCR products to ensure the sequence quality from both directions. Reverse transcription-PCR (RT-PCR) was conducted using the OneTaq one-step RT-PCR kit (NEB, USA), according to the manufacturer’s protocol. The PCR products were separated by electrophoresis using 1.5% agarose gels. The RT-PCR products were excised from the gel and purified using the QIAquick PCR purification kit (Qiagen, USA); DNA sequencing was performed on the purified PCR products using the BigDye Terminator cycle sequencing v3.1 kit (ABI, Foster City, CA) on an ABI 3500 genetic analyzer (Applied Biosystems, Life Technologies, CA, USA). Sequencing of each PCR product was performed from both sides. The majority of the sequences contain unique peaks with quality values greater than 40. The obtained sequences were aligned with other H5 avian influenza virus strains from GenBank (Clustal W algorithm), and phylogenetic trees were constructed using the neighbor-joining method and the Tamura-Nei model (7) with MEGA 7.0 software (8), with 1,000 bootstrap replications to automatically assign confidence levels for the branches.

BLASTn analyses showed significant genetic similarity of the chicken isolate in all eight genes to the highly pathogenic H5 influenza viruses isolated from poultry in the Middle East and West Africa (Table 1) (9) that caused outbreaks there. The HPAI virus status was confirmed by H5 cleavage site sequence, directly from clinical specimens. The cleavage site motif was identified in A/chicken/North Kazakhstan/184/2020 as KRRKR/GLF, identical to that found in other 2016–2017 European viruses, including other UK poultry cases (2).

**TABLE 1:** Comparison of the nucleotide sequences of all genes of strain influenza A/chicken/North Kazakhstan/184/2020 (H5N8) with those of its closest genetic relatives in GenBank

Gene or segment	Size (nucleotides)	GC content (%)	Closest relative	Identity at nucleotide level (%)	GenBank accession no. for the closest relative
PB2	2,280	44.78	A/mule_duck/France/20335/2020 (H5N8)	99.43	MZ166257.1
PB1	2,341	43.57	A/chicken/Kazakhstan/23/2020 (H5N8)	99.39	ON943052.1
PA	2,233	44.04	A/chicken/Nigeria/VRD21-53B_21VIR2288-5/2021 (H5N8)	99.73	MW961481.1
HA	1,777	41.98	A/chicken/Kazakhstan/23/2020 (H5N8)	99.66	ON943054.1
NP	1,565	47.03	A/environment sample/China/TZ001/2021 (H5N8)	99.28	OL442771.1
NA	1,461	44.21	A/Chlidonias hybrida/Hubei/55/2020 (H5N8)	99.29	MW505353.1
M	1027	48.98	A/poultry/Benin/21-A-08-035-O/2021 (H5N1)	99.38	ON870414.1
NS	851	43.01	A/mallard/Shanghai/JDS20876/2020 (H5N8)	99.29	MW362177.1

**FIG 1:**
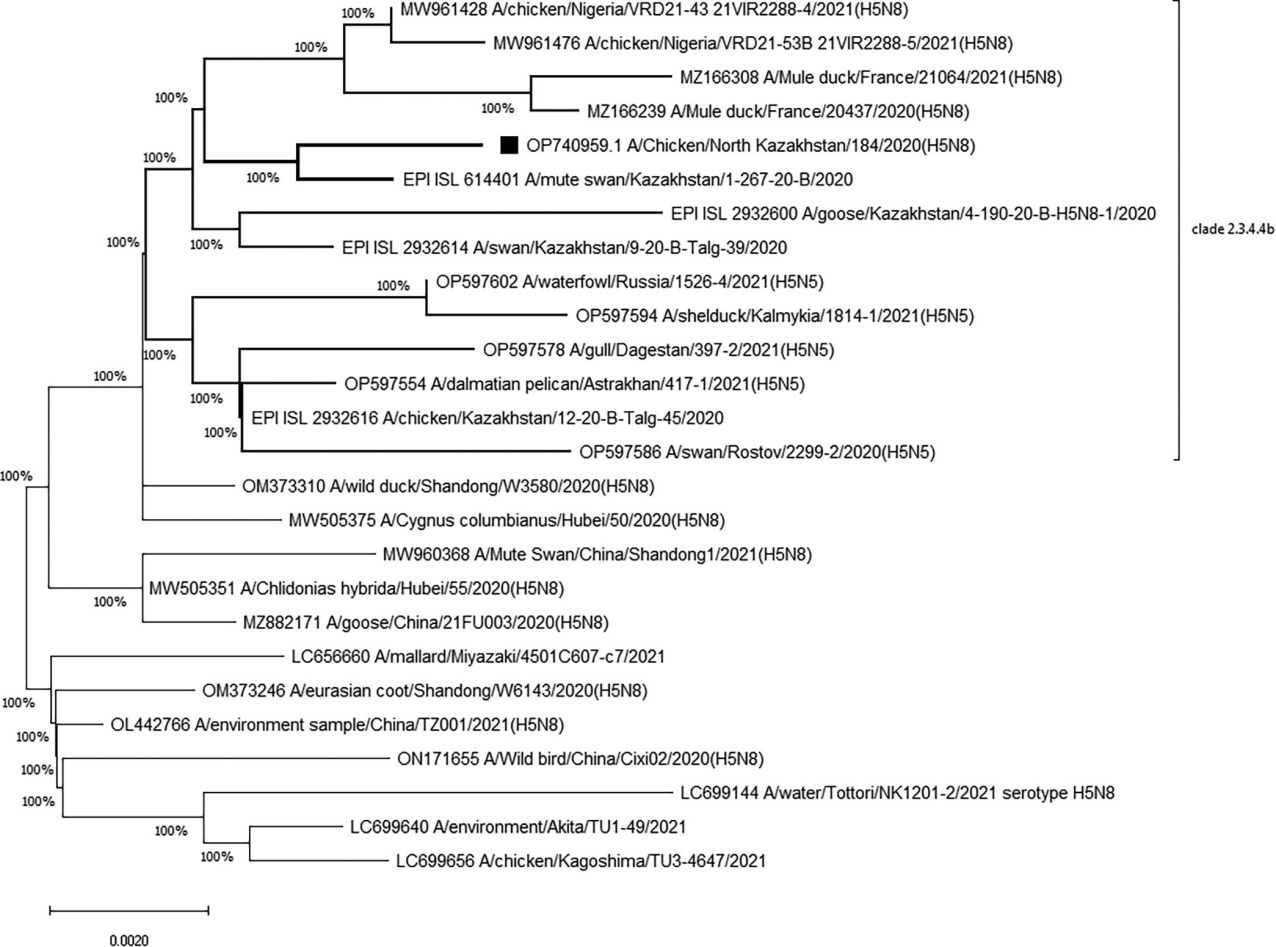
Phylogenetic tree of the HA gene of influenza A/H5N8 viruses of clade 2.3.4.4b and other viruses circulating around the globe.

HPAI virus subtype H5N8 strains circulating among domestic and feral birds in Eurasia and Africa between 2020 and 2021 were selected for phylogenetic analysis. In the phylogenies, the HA gene of virus A/chicken/North Kazakhstan/184/2020 clustered with the subtype H5N8 viruses detected among poultry and wild birds in Russia, China, and Europe. The phylogenetic tree of the HA gene shows that the strain from this study, as a previously described strain from Kazakhstan (GISAID reference number EPI ISL 2932614) (3), belongs to the H5 clade 2.3.4.4b of HPAI viruses consisting of isolates from the Middle East and West Africa (Fig. 1). Other closely related prototypes were from Eastern Europe. The Central Asia Migratory Flyway passes over Kazakhstan, and birds have intermediate stops in Northern Kazakhstan. It has been suggested that the A/H5N8 subtype was introduced by migrating birds.

### Data availability.

The complete genome sequence of A/chicken/North Kazakhstan/184/2020 is available at GenBank under the accession numbers OP740956 to OP740959. The raw sequence reads were deposited under BioProject accession number PRJNA944784.
